# Scalable and Ultra‐Sensitive Nanofibers Coaxial Yarn‐Woven Triboelectric Nanogenerator Textile Sensors for Real‐Time Gait Analysis

**DOI:** 10.1002/advs.202401436

**Published:** 2024-05-15

**Authors:** Yihan Wang, Lang Chu, Si Meng, Mingxuan Yang, Yidan Yu, Xiaokang Deng, Cheng Qi, Tiantian Kong, Zhou Liu

**Affiliations:** ^1^ Department of Biomedical Engineering School of Medicine Shenzhen University Shenzhen Guangdong 518000 China; ^2^ College of Chemistry and Environmental Engineering Shenzhen University Shenzhen Guangdong 518000 China; ^3^ Guangdong Provincial Key Laboratory of Micro/Nano Optomechatronics Engineering College of Mechatronics and Control Engineering Shenzhen University Shenzhen Guangdong 518000 China; ^4^ Department of Urology Shenzhen Institute of Translational Medicine The First Affiliated Hospital of Shenzhen University Shenzhen Second People's Hospital Shenzhen Guangdong 518037 China

**Keywords:** biomedical applications, coaxial yarns, electrospinning, triboelectric nanogenerators, wearable devices

## Abstract

Yarn‐woven triboelectric nanogenerators (TENGs) have greatly advanced wearable sensor technology, but their limited sensitivity and stability hinder broad adoption. To address these limitations, Poly(VDF‐TrFE) and P(olyadiohexylenediamine (PA66)‐based nanofibers coaxial yarns (NCYs) combining coaxial conjugated electrospinning and online conductive adhesive coating are developed. The integration of these NCYs led to enhanced TENGs (NCY‐TENGs), notable for their flexibility, stretchability, and improved sensitivity, which is ideal for capturing body motion signals. One significant application of this technology is the fabrication of smart insoles from NCY‐TENG plain‐woven fabrics. These insoles are highly sensitive and possess antibacterial, breathable, and washable properties, making them ideal for real‐time gait monitoring in patients with diabetic foot conditions. The NCY‐TENGs and their derivatives show immense potential for a variety of wearable electronic devices, representing a considerable advancement in the field of wearable sensors.

## Graphical abstract

By employing a novel preparation method combining coaxial conjugated electrospinning and online conductive adhesive coating, this study have created flexible and sensitive yarn‐woven triboelectric nanogenerators (TENGs using P(olyadiohexylenediamine‐based and Poly(VDF‐TrFE)‐based nanofibers coaxial yarns. These TENGs are suitable for smart insoles, offering high sensitivity, antibacterial, breathable, and washable properties, ideal for real‐time gait monitoring in diabetic patients.

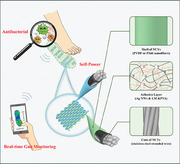

## 1 Introduction

Triboelectric nanogenerators (TENG) continuously capture energy from ambient mechanical movements through the triboelectric effect and electrostatic induction.^[^ ^1^, ^2^, ^3^ ^]^ Their structural simplicity is ideal for lightweight, miniaturized, and wearable sensor applications.^[^ ^4^, ^5^, ^6^, ^7^ ^]^ Textile‐like TENG wearable sensors are naturally compatible with regular clothing, capable of converting mechanical or electrical stimuli into electrical outputs, providing non‐invasive monitoring of various physiological parameters.^[^ ^8^, ^9^, ^10^, ^11^, ^12^ ^]^ TENG sensors generally divided into two types based on their basic structural unit: fabric‐assembled TENG and yarn‐woven TENG.^[^ ^13^, ^14^, ^15^, ^16^, ^17^, ^18^ ^]^ The former is developed by adhering two fabrics with distinct triboelectric properties onto a flat electrode, while the latter contains a conductive core encased by a triboelectric shell woven into the TENG textiles. Yarn‐woven TENGs, marked by their core‐shell configuration, offer unique benefits like flexibility, breathability, and the ability to integrate seamlessly with conventional fabrics.^[^ ^19^, ^20^, ^21^, ^22^ ^]^

The composition unit of yarn‐woven TENG is coaxial fibers. The performance of yarn‐woven TENG is intrinsically tied to the production technique of coaxial fibers.^[^ ^23^ ^]^ A variety of methods, such as coaxial spinning, coating, film‐wrapping, and yarn‐wrapping have been developed for coaxial‐fiber TENGs.^[^ ^24^, ^25^, ^26^ ^]^ In the coaxial spinning, the inner conductive and outer triboelectric fluids are extruded into the coagulation bath to form coaxial fibers.^[^ ^27^, ^28^, ^29^, ^30^, ^31^ ^]^ The coating method involves applying the triboelectric material processing fluid to a conductive fiber as a substrate.^[^ ^32^, ^33^, ^34^, ^35^, ^36^ ^]^ In the film and yarn wrapping techniques, conductive fibers are wrapped with film‐like or fibrous triboelectric materials, respectively.^[^ ^37^, ^38^, ^39^, ^40^, ^41^, ^42^ ^]^ However, TENG sensors made through these methods often have limited contact areas between yarns, reducing the triboelectric charge density and overall output, which leads to lower sensitivity. Also, mismatches between conductive fibers and triboelectric materials can result in weak bonds between the core and the shell. This can cause an uneven distribution of the shell material, increased resistance at the junction, and compromised mechanical integrity of the yarn. When subjected to external forces, the core and shell may shift relative to each other, affecting the core‐shell structure and causing resistance variations in the yarn. Uneven distribution of the triboelectric materials could also lead to inconsistent charge generation and potential charge leakage. These sensitivity and stability issues undermine the ability of TENG sensors to reliably detect subtle stresses, crucial for real‐time health monitoring.

The electrospinning method can enhance the sensitivity of TENG sensors by increasing effective frictional contact areas and boosting charge production.^[^ ^43^, ^44^, ^45^, ^46^ ^]^ Nanofibers coaxial yarns (referred to as NCYs) can be fabricated by electrospinning in a one‐step or two‐step process for building TENGs, also referred as NCY‐TENGs. The two‐step method involves constructing a nanofiber mat, which is then wrapped around the core electrode fiber. For instance, a P(olyadiohexylenediamine (PA66) nanofiber mat and a poly(VDF‐TrFE) (abbreviated as PVDF) anofiber mat are created by electrospinning and subsequently wrapped onto conductive wires.^[^ ^47^ ^]^ On the other hand, the one‐step method encases the core fiber directly with nanofibers, making it more suitable for large‐scale production. For instance, a novel coaxial conjugated electrospinning is used to coat PVDF nanofibers on conductive core fibers including silver‐plated nylon, stain‐steel, and CNT yarns, resulting in lightweight NCYs.^[^ ^48^, ^49^, ^50^ ^]^ Two distinct NCYs can be interwoven into a high‐performing NCY‐TENG. The ultra‐sensitive NCY‐TENGs with nanoscale roughness, coupled with a machine learning model, allow for real‐time identification of sensing materials. These developments highlight that nanofibers yarns can amplify the electrical output and sensitivity of yarn‐woven TENG sensors. However, achieving robust mechanical bonding of the core and shell remains a challenge. Despite the demonstrated versatility of electrospinning for various TENG designs, the application of these enhanced NCY‐TENGs and their derivative textiles in biomedical scenarios, which have additional requirements like being antibacterial, breathable, and washable, remains largely unexplored.^[^ ^16^, ^20^, ^37^, ^47^ ^]^

**Figure 1:**
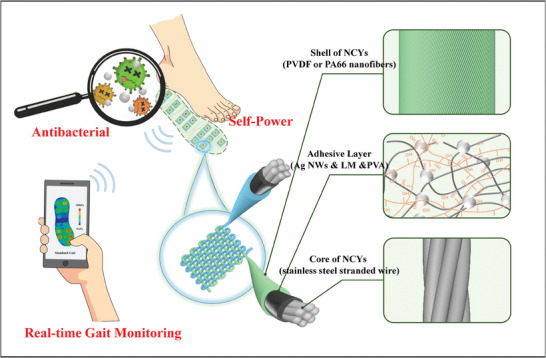
Schematic diagram of a yarn‐woven TENG textile based on NCYs and its application in gait monitoring.

In this work, we developed a scalable, ultra‐sensitive, and stable yarn‐woven TENG textile based on NCFs. This textile is specially designed for biomedical applications, including diabetic foot gait monitoring, as shown in **Figure** **1**. The novel textile is achieved by synergistically combining coaxial conjugated electrospinning with an online conductive adhesive coating process. We fabricated NCYs by electrospinning PVDF and PA66 nanofibers onto stainless steel strands. These strands were pre‐coated with a polyvinyl alcohol (PVA) adhesive containing silver nanowires (Ag NWs) and liquid metals (LM), which enhances both conductivity and functionality. Our approach effectively addresses the common challenges of sensitivity and stability in yarn‐woven TENGs through a combination of advanced nanofiber architecture, a carefully screened triboelectric shell, and the integration of conductive adhesive. The addition of Ag NWs not only increases conductivity but also offers antibacterial properties, improving the textiles wearability. The resultant NCYs exhibit exceptional flexibility, mechanical strength, sewing capability, and braiding capacity, enabling their incorporation into various textile designs. By employing flexible textile structures like braiding, weaving, and knitting, TENGs textiles made from NCYs demonstrate varying degrees of flexibility, stretchability, and sensing sensitivity. For instance, ribbed fabrics offer increased stretchability, whereas plain‐woven fabrics provide superior sensitivity. Owing to their scalable preparation, controllable structure, comfortable wearability, and functional expansibility, these NCY‐TENGs textiles hold great potential for real‐time monitoring of body motion signals, such as gait and joint activity in diabetic foot patients. Additionally, they show promising prospects in the broader field of wearable electronic devices.

## 2 Results

To fabricate two distinct coaxial nanofibers yarns for TENG assembly, we employed coaxial conjugated electrospinning to deposit PA66 and PVDF nanofibers onto stainless steel strands. These yarns have a core‐shell structure, where the core consists of highly conductive and sturdy stainless steel strands, while the shell is made of either PA66, which exhibits positive triboelectric properties, or PVDF, known for its negative triboelectric properties. These strands are composed of individual filaments, each with a diameter of only 12 µm. Due to their exceptionally fine composition, these stainless‐steel strands exhibit significant flexibility, a property that has facilitated their adoption within the textile industry as materials for sewing threads. The resulting TENG, assembled from two coaxial yarns with functional shells of PA66 and PVDF nanofibers, is referred as NCY‐TENGs. The selection of PVDF and PA66 was based on their exceptional spinnability and pronounced electron affinity differences. These properties are crucial as they enable efficient charge transfer and enhance frictional charge generation during the contact‐separation process inherent in TENG operation. The yarn's efficient charge generation ability ensures the production of substantial electrical signals even under minimal external forces, thereby improving the signal‐to‐noise ratio and enhancing the TENG's overall sensing sensitivity. Furthermore, PVDF's piezoelectric properties allow for even greater charge generation efficiency compared to other TENG materials. The nanoscale topography of functional shells of NCYs increases the contact area between yarns, thus enhancing frictional charge generation. This combination of material selection and nanoengineering is crucial for optimizing the TENG efficiency.

**Figure 2:**
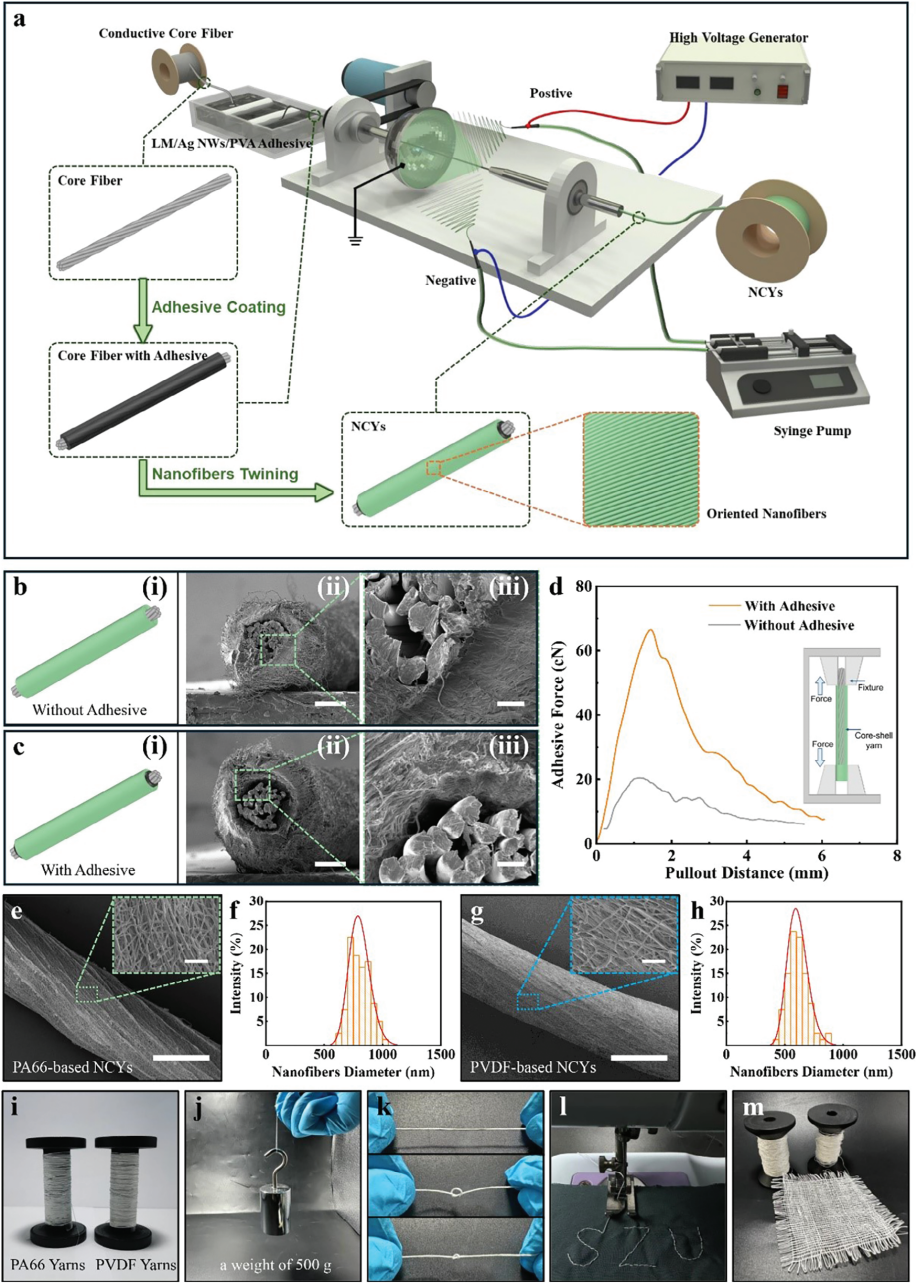
Preparation process, morphology, properties and optical photographs of PA66‐based and PVDF‐based NCYs. a) Schematic diagram depicting the preparation process of PA66‐based and PVDF‐based NCYs; b) Structural schematic diagram (left) and SEM images (middle and right) of PA66‐based NCYs without adhesive, scale bar: 100 µm (middle) and 20 µm (right); c) Structural schematic diagram (left) and SEM images (middle and right) of PA66‐based NCYs with adhesive, scale bar: 100 µm (middle) and 20 µm (right); d) Comparison of adhesive force between shell and core of NCYs with/without adhesive; e) SEM images of the surface of PA66‐based NCYs, scale bar: 400 µm. The inserted image is a partial magnification of the PA66‐based NCYs, scale bar: 20 µm; f) Diameter distribution of nanofibers in the shell of PA66‐based NCYs; g) SEM images of the surface of PVDF‐based NCYs, scale bar: 400 µm. Inserted image is a partial magnification of the PVDF‐based NCYs, scale bar: 20 µm; h) Diameter distribution of nanofibers in the shell of PVDF‐based NCYs; (i) Optical photograph of PA66‐based and PVDF‐based NCYs; j) A 10 cm segment of PA66‐based NCY supporting a weight of 500 g; k) PA66‐based NCY being knotted; l) PA66‐based NCY being sewn onto a standard cotton textile with a “SZU” pattern; m) PA66‐based and PVDF‐based NCYs were woven into a composite fabrics.

We have integrated the coaxial conjugated electrospinning with the on‐line coating of conductive adhesives into a single‐step, continuous, and batch production process, as shown in **Figure** **2**a and Figure S1 and Movie S1 (Supporting Information). A common issue with NCYs is the poor bonding between the conductive core strands and the polymer nanofiber shells, leading to interface gaps. These gaps increase contact resistance, causing the nanofiber shells to slip and delaminate from the conductive core metal strands. This weakens the electrical signal output and reduces the stability of the sensing system. To address this issue, we have introduced a novel conductive adhesive, consisting of liquid metal, Ag NWs, and PVA (known for its adhesive properties), as shown in Figure S2 (Supporting Information). The high conductivity and fluidic nature of liquid metal allows it to fill irregular spaces between the core and shell effectively, thus reducing contact resistance. Incorporating Ag NWs within the PVA adhesive creates a 3‐D conductive network, which not only enhances conductivity by connecting the liquid metal droplets but also exhibits antibacterial properties, reducing the risk of infection in potential biomedical applications. This conductive adhesive significantly improves the integration between the NCYs shell and core, enhancing adhesion, as illustrated in Figure 2b,c and Figure S3 (Supporting Information). Remarkably, this conductive adhesive triples the adhesive force between the shell and core metal strand, compared to fibers without it, as shown in Figure 2d. The specific orientation, uniformity, and tight encapsulation of the metal core strands by the nanofiber‐based shell layers are crucial for enhancing the NCYs performance. These coaxial yarns are featured by their uniform structure, including shell layers with a narrow range of nanofiber diameters and consistent thickness. The uniformly nanofibers in the shell layers are spirally orientated, as shown in Figure 2e–h. This demonstrates the precision and consistency of our fabrication process, with such fine microstructures being essential for enhancing the charge production efficiency of the NCYs.

This coaxial conjugate electrospinning process of NCYs is continuous and scalable, and has been used to prepare PA66‐based and PVDF‐based NCYs in large quantities, as shown in Figure 2i. These NCYs are highly durable and remarkably flexible due to their robust and flexible core strands. For instance, a 10 cm segment of PA66‐based NCYs can support a weight of 500 g, ≈10 000 times of its own weight, as shown in Figure 2j; They also exhibit exceptional flexibility, easily resisting bending and knotting, as demonstrated in Figure 2k. These properties make NCYs compatible with commercial sewing equipment. They can be smoothly integrated into intricate patterns on standard cotton textiles, as shown in Figure 2l. Moreover, these fibers can be easily woven into fabrics, demonstrating their compatibility with existing textile manufacturing processes, as illustrated in Figure 2m.

**Figure 3:**
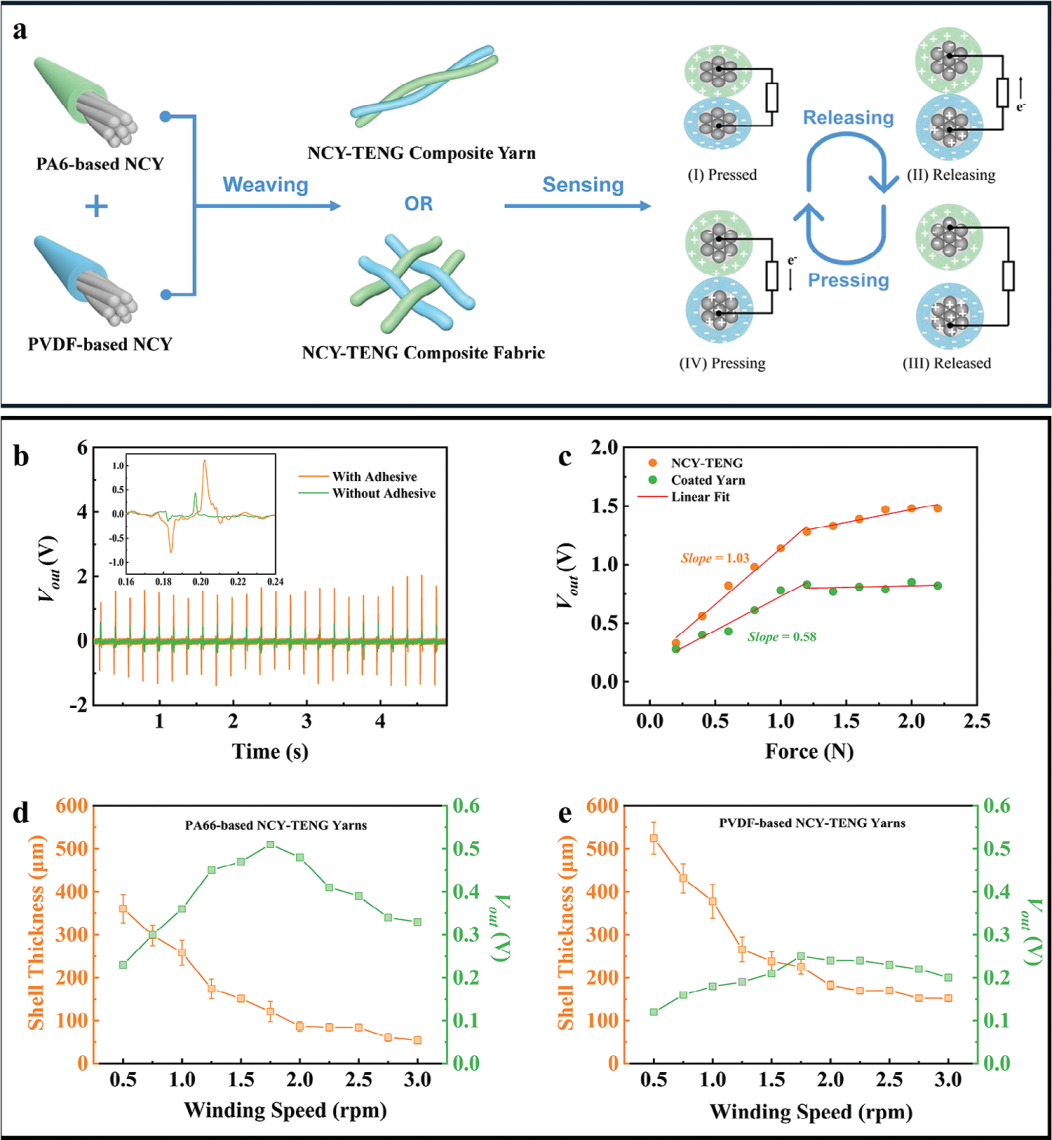
Electrical signal generation mechanism and properties of composite textiles based on PA66‐based and PVDF‐based NCYs. a) Schematic diagram of electrical signal generation mechanism of NCY‐TENG composite yarns and NCY‐TENG composite fabrics; b) Comparison of electrical signals generated by NCY‐TENG composite yarns with/without adhesive under the same stress conditions; c) Comparison of electrical signals generated by NCY‐TENG composite yarns and coated composite yarns; d) Comparison of output voltage generated by the contact of PA66‐based NCYs with varying shell thicknesses and PVDF‐based NCYs with ≈350 µm shell thickness under the same stress conditions; e) Comparison of output voltage generated by the contact of PVDF‐based NCYs with varying shell thicknesses and PA66‐based NCYs with ≈350 µm shell thickness under the same stress conditions.

TENG sensors can be devised by twisting or weaving PA66‐based and PVDF‐based NCYs, as shown in **Figure** **3**a. These devices generate electrical signals through the Contact–Separation (C–S) mode. When external pressure is applied, the PVDF‐based and PA66‐based NCYs come into contact, initiating a charge transfer due to the differing polarities of their triboelectric material shells. The PVDF shell gains electrons, lowering its electric potential, while the PA66 shell loses electrons, increasing its potential. This contact results in maximum charge exchange, balancing the charge distribution and temporarily halting current generation. When the external force is released, the yarns contact area shrinks, preventing immediate redistribution of the initial charges and causing excess charges to accumulate on both yarns surface. To balance these excess charges, electrons transfer from PVDF‐based NCYs electrodes to the PA66‐based NCYs electrodes, creating an induced current in the connected external circuit. Once the external force is fully released, the contact area stabilizes, and charge transfer through the external circuit reaches equilibrium, stopping new current generation. When external pressure is reapplied, this equilibrium is disrupted, the contact area between the yarns expands, and the potential difference increases. Electrons move from the PA66‐based NCYs electrodes to PVDF‐based NCYs electrodes, reducing the potential difference. This cycle of contact and separation generates an alternating current in the TENG sensor, a process that repeats with each application and release of external pressure.

We conducted a series of experiments to evaluate the impact of a conductive adhesive and a nanofiber structure in the shell layer of NCYs on charge production efficiency and sensing sensitivity. Initially, we prepared two sets of NCYs: one set incorporated the conductive adhesive and the other did not. These yarns were used to assemble TENG sensing devices. When tested under identical stress conditions (2 N, 5 Hz), the devices with the conductive adhesive showed a significant performance boost. The output voltage (*V*_out_) of these devices was ≈241% higher compared to those without the adhesive, as shown in Figure 3b. Additionally, we compared TENG sensing devices made from NCYs nanofiber microstructures to devices made from conventional coated fibers without such microstructures. Remarkably, the devices using NCYs with nanostructures consistently exhibited significantly higher *V*_out_ under various stress conditions, compared to the conventional devices, as shown in Figure 3c. The enhanced sensing sensitivity of TENG sensing devices using NCYs can be quantified using the following equation:

(1) $$K=\frac{\Delta V_{\mathrm{out}}}{\Delta \sigma }$$

Where *K* represents sensitivity coefficient; Δ*V*_out_ represents the change in output voltage generated by the sensor subjected to force; Δσ represents the force applied to the yarn‐like sensor or the pressure applied to the fabric‐like sensor. Through the calculations, we found that the sensing factor of devices assembled with NCYs is nearly double that of devices made with ordinary coated fibers. This significant increase in sensitivity indicates that adding nanostructures to the surface of NCYs and conductive adhesives between their core and shells significantly enhances their responsiveness and overall performance.

Optimizing the shell thickness of NCYs is crucial for enhancing their performance. If the shells are too thin, they risk charge leakage which reduces efficiency. Conversely, extremely thick non‐conductive shells can increase internal resistance, impeding efficient charge transfer. To determine the optimal shell thickness for sensing performance, we adjust the winding speed to fine‐tune the shell thickness of PA66‐based and PVDF‐based NCYs. The result shows that a lower winding speed results in thicker shells. PA66‐based NCYs with varying shell thicknesses and PVDF‐based NCYs with ≈350 µm shell thickness were assembled to create twisted yarn‐like TENG. Similarly, PVDF‐based NCYs with varying shell thicknesses and PA66‐based NCYs with ≈350 µm shell thickness were assembled to create twisted yarn‐like TENG. These twisted yarn like TENGs were used to investigate how the shell thickness of NCYs affects the response voltage under consistent stress conditions, and to optimize the optimal shell thickness for PA66‐based NCYs and PVDF‐based NCYs. For both yarn types, there is an initial increase in response voltage with increasing shell thickness, followed by a decrease, as shown in Figure 3d,e. The optimal output voltage is achieved at a shell thickness of 120 µm for PA66‐based NCYs and 225 µm for PVDF‐based NCYs. These findings highlight the critical role of shell thickness in maximizing NCYs efficiency of NCYs. Therefore, we have chosen NCYs with these specific thicknesses (PA66‐based NCYs: 120 µm; PVDF‐based NCYs: 225 µm) for subsequent experiments.

**Figure 4:**
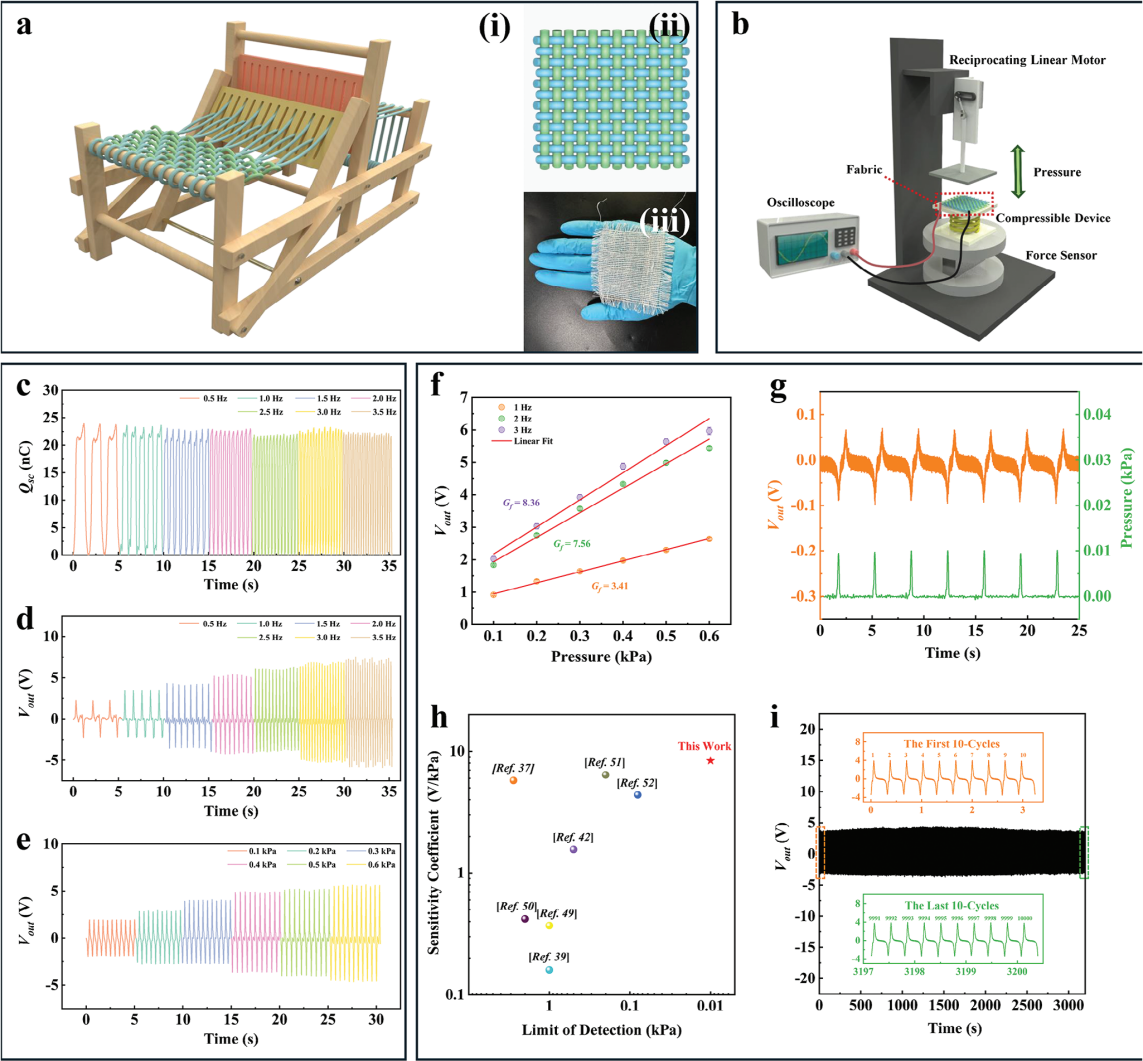
Preparation process, structure, performance testing system and properties of PA66/PVDF‐based NCY‐TENG plain‐woven fabrics. a) Schematic diagram of preparation process (left) and structure (upper right) and optical photograph (lower right) of PA66/PVDF‐based NCY‐TENG plain‐woven fabrics; b) Schematic diagram of performance testing system for PA66/PVDF‐based NCY‐TENG plain‐woven fabrics periodic stress sensing testing; c) Comparison of the amount of charge transfer generated by PA66/PVDF‐based NCY‐TENG plain‐woven fabrics under 0.6 kPa pressure at different frequencies; d) Comparison of the output voltage generated by PA66/PVDF‐based NCY‐TENG plain‐woven fabrics under 0.6 kPa pressure at different frequencies; e) Comparison of the output voltage generated by PA66/PVDF‐based NCY‐TENG plain‐woven fabrics under the different pressure at 2 Hz; f) Linear fitting of open circuit voltage generated by PA66/PVDF‐based NCY‐TENG plain‐woven fabrics under different pressure conditions; g) Open circuit voltage generated by PA66/PVDF‐based NCY‐TENG plain‐woven fabrics under a periodic pressure of 0.01 kPa at a 0.3 Hz; h) Comparison of sensitivity and detection limits among PA66/PVDF‐based NCY‐TENG plain‐woven fabrics and other reported TENG sensing fabrics based on coaxial fibers.^[^ ^37^, ^39^, ^42^, ^49^, ^50^, ^51^, ^52^ ^]^ Notes: Some of the values in these references were not directly provided, were estimated based on the graph; i) The open circuit voltage generated by PA66/PVDF‐based NCY‐TENG plain‐woven fabrics under long‐term periodic pressure.

To simulate real‐life scennaris for wearables, we investigate the performance of NCYs‐TENGs under conditions that mimic human walking. We wove and integrated these yarns into plain‐woven fabric, a material typically used for insoles, as shown in **Figure** **4**a. A system equipped with reciprocating motors was developed to mimic the effect of a foot impact on insoles during walking. These motors exert periodic pressure on the NCYs, mimicking the dynamics of human steps, as demonstrated in Figure 4b and Figure S4 (Supporting Information).

To confirm our sensing fabrics suitability for gait monitoring, we assessed its ability to detect the force frequencies and magnitudes typical in human movement. Walking and running generally involve frequencies between 1 and 3 Hz, equivalent to 60 and 180 steps per minute. We tested the NCY‐TENG plain‐woven fabric under these conditions, specifically measuring its short‐circuit transferred charge (*Q*_sc_), *V*_out_, and short‐circuit charge transfer (*I*_sc_) at a periodic stress of 0.6 kPa, as shown in Figure 4c,d and Figures S5 and S6. We found that the fabric generated stable and pronounced electrical signals within a frequency range of 0.5–3.5 Hz, covering the typical gait frequencies. Notably, higher frequencies of pressure resulted in a greater *V*_out_ in the fabric. Furthermore, the *V*_out_ of the sensing fabric also increased with the pressure under a constant frequency of 2 Hz, as shown in Figure 4e. These results demonstrate that the fabric can monitor plantar stress in real‐time by measuring the voltage response to varying pressures, thus meeting the requirements of gait analysis.

To evaluate the effectiveness of our NCY‐TENG plain‐woven fabrics in human activity monitoring, we examined the correlation between their sensitivity and the frequency of applied stress. Generally, TENG yarns and fabrics are highly sensitive at high frequencies. However, our NCY‐TENG plain‐woven fabric demonstrates a remarkable sensitivity coefficient up to 8.36 V kPa^−1^ even at a low frequency of 3 Hz, as shown in Figure 4f. This indicates a high sensitivity to subtle movements. Furthermore, the NCY‐TENG plain‐woven fabrics are proficient at detecting extremely low stress levels and at ultra‐low frequencies. For instance, they can generate stable and high‐quality electrical signals, with a strong signal‐to‐noise (S/N) ratio, under mild conditions such as a periodic ultra‐low pressure of 0.01 kPa at a frequency of just 0.3 Hz (see Figure 4g). This sensitivity and detection limit surpass most other fiber‐type or fabric‐type TENG sensing devices, as demonstrated in Figure 4h. Additionally, we tested the durability and stability of the NCY‐TENG plain‐woven fabrics. We exposed the fabric to a cyclic stress of 0.4 kPa at a frequency of 3 Hz for 10 000 cycles. The difference in open circuit voltages between the last ten cycles and the initial ten cycles is less than 1%, as shown in Figure 4i. This high sensitivity, ability to monitor low stress levels, and performance stability over time make the NCY‐TENG plain‐woven fabrics ideal for long‐term, comprehensive, and accurate monitoring of human activities.

**Figure 5:**
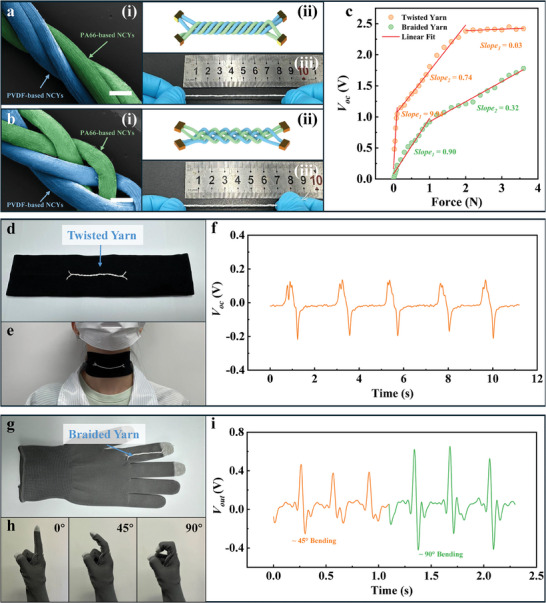
Morphology, structure, properties and applications of PA66/PVDF‐based NCY‐TENG composite yarns. a) Pseudo‐color processed SEM image (Left), structural model diagram (upper right) and optical photograph of PA66/PVDF‐based NCY‐TENG twisted yarn. Scale bar: 500 µm; b) Pseudo‐color processed SEM image (Left), structural model diagram (upper right) and optical photograph of PA66/PVDF‐based NCY‐TENG braided yarn. Scale bar: 500 µm; c) Linear fitting of open circuit voltage generated by PA66/PVDF‐based NCY‐TENG twisted yarns and PA66/PVDF‐based NCY‐TENG braided yarns under different stress conditions; d) Optical photograph of the smart neck‐band incorporated PA66/PVDF‐based NCY‐TENG twisted yarn; e) Optical photograph of the smart neck‐band used to monitor swallowing movements; f) The output voltage signal generated by the smart neck‐band during monitoring swallowing movements; g) Optical photograph of the smart glove incorporated PA66/PVDF‐based NCY‐TENG braided yarn; h) Optical photograph of the smart glove used to monitor finger bending movements; i) The output voltage signal generated by the smart glove during monitoring finger bending movements.

To enhance the versatility of NCYs in wearable devices, we have also explored incorporating them into various textile structures. This includes not only plain‐woven fabrics but also twisted yarn, braided yarn, and ribbed fabric. NCY‐TENG twisted and braided yarns were made through twisting and weaving, respectively, as shown in **Figure** **5**a,b. Interestingly, these yarns, despite originating from the same fibers, exhibit distinct mechanical and sensing properties. For instance, under low stress, NCY‐TENG twisted yarns show a higher sensing sensitivity compared to the braided ones. However, the braided yarns can detect a broader stress range, as shown in Figure 5c. This difference is mainly attributed to the looser circular weaving of the NCYs in the braided yarn. Under low stress, this structure results in a smaller fiber contact area, reducing charge transfer efficiency. Yet, it allows the effective contact area to increase progressively with rising stress over a wider range. This weaving method endows the textiles made from non‐stretchable NCYs with stretchability. Therefore, we can tailor yarns with specific characteristics, expanding their suitability for various wearable devices. Given the unique performance attributes of NCY‐TENG twisted and braided yarns, we customized their application for specific body motion monitoring tasks. The NCY‐TENG twisted yarn, known for its high sensitivity and lightweight, is ideal for monitoring subtle movements like swallowing in the neck area, as illustrated in Figure 5d‐f. The stretchable NCY‐TENG braided yarn, on the other hand, is suitable for tracking dynamic movements, such as joint activity, especially in smaller joints like fingers, as shown in Figure 5g,i. Therefore, by integrating NCY‐TENG twisted and braided yarns into common accessories like neck‐bands and gloves, we can transform them into devices capable of monitoring and precisely measuring activities like swallowing and finger bending.

**Figure 6:**
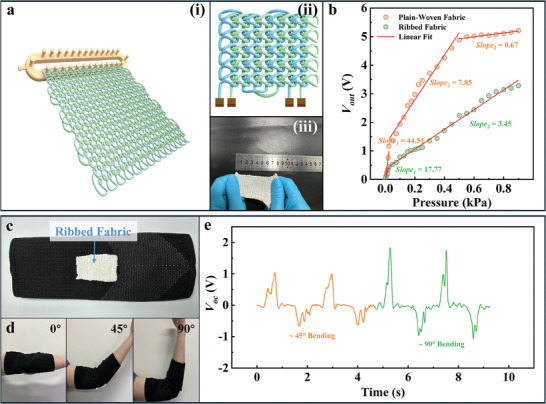
Preparation process, structure, properties and applications of PA66/PVDF‐based NCY‐TENG ribbed fabrics. a) Schematic diagram of preparation process (left) and structure (upper right) and optical photograph (lower right) of PA66/PVDF‐based NCY‐TENG ribbed fabrics; b) Linear fitting of open circuit voltage generated by PA66/PVDF‐based NCY‐TENG plain‐woven fabrics and PA66/PVDF‐based NCY‐TENG ribbed fabrics under different pressure conditions; c) Optical photograph of the smart elbow protector incorporated PA66/PVDF‐based NCY‐TENG ribbed fabric; d) Optical photograph of the smart elbow protector used to monitor elbow bending movements; f) The output voltage signal generated by the smart elbow protector during monitoring elbow bending movements.

We further expanded the application of NCYs by fabricating NCY‐TENG ribbed fabrics through knitting and integrating them into everyday wearable items, as shown in **Figure** **6**a. These ribbed fabrics have a unique structure that results in distinct performance characteristics compared to NCY‐TENG plain‐woven fabrics. Specifically, NCY‐TENG ribbed fabrics have a wider linear detectable pressure range, although their sensing sensitivity is slightly lower than that of woven fabrics, as shown in Figure 6b. This performance difference is partially due to their loose circular weaving structure, similar to that in NCY‐TENG braided yarns. The stretchability of the NCY‐TENG ribbed fabric makes it ideal for monitoring movements in larger joints like the elbow. We utilized this feature by incorporating these ribbed fabrics into commercial elbow protectors, as shown in Figure 6c, transforming them into smart wearables that can precisely track elbow movement. As such, the modified elbow protector can accurately perceive the elbow bending at various angles, as demonstrated in Figure 6d,e.

**Figure 7:**
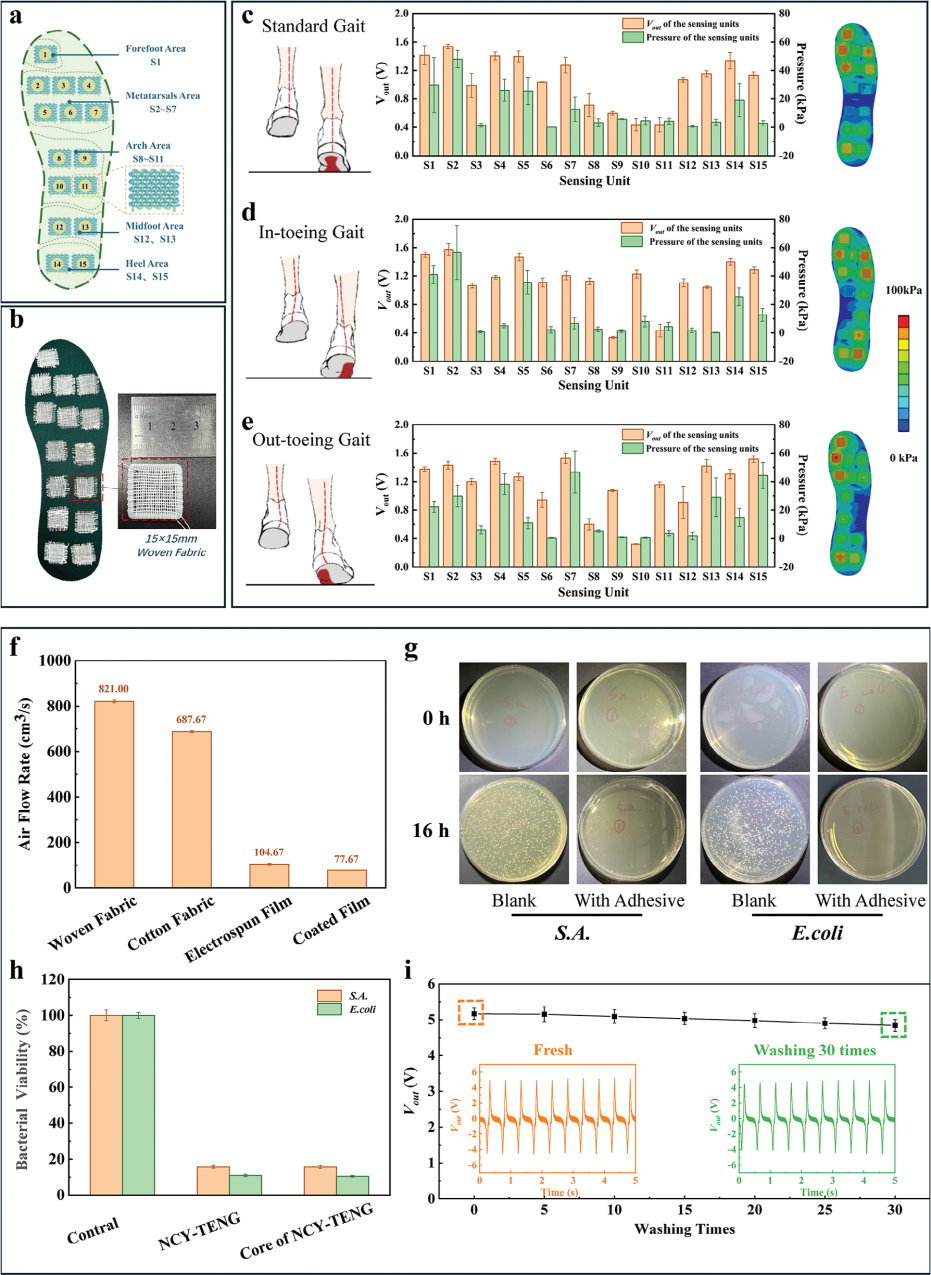
The PA66/PVDF‐based NCY‐TENG plain‐woven fabrics were used to make smart insoles for gait monitoring and their service performance. a) Layout diagram of PA66/PVDF‐based NCY‐TENG woven fabrics in smart insole; b) Optical photograph of the smart insole incorporated PA66/PVDF‐based NCY‐TENG plain‐woven fabric; c‐e) Schematic diagrams of posture and stress area for standard (Figure c, left), in‐toeing (Figure d, left), and out‐toeing (Figure e, left) gait. The output voltages and calculated pressure values of the PA66/PVDF‐based NCY‐TENG plain‐woven fabrics in various areas of smart insoles under standard (Figure c, middle), in‐toeing (Figure d, middle), and out‐toeing (Figure e, middle) gait. Simulation diagrams of the pressure distribution on each area of the smart insole under standard (Figure c, right), in‐toeing (Figure d, right), and out‐toeing (Figure e, right) gait. f) Comparison of breathability among PA66/PVDF‐based NCY‐TENG plain‐woven fabric and other fabrics commonly used for wearable devices; g) Colony growth of Staphylococcus aureus and Escherichia coli co‐cultured with PA66/PVDF‐based NCY‐TENG plain‐woven fabric; h) The bacterial viability of Staphylococcus aureus and Escherichia coli co‐cultured with PA66/PVDF‐based NCY‐TENG plain‐woven fabric and core of NCY‐TENG yarn containing adhesive; i) Open circuit voltage generated by PA66/PVDF‐based NCY‐TENG woven fabrics washed for different times. Insert is the open circuit voltage signal generated by fresh fabric (orange) and fabric washed 30 times (green) under periodic pressure.

As a proof of concept, we developed insoles for gait monitoring. Based on our investigations of various textile structures, we selected NCY‐TENG plain‐woven fabrics with their superior sensing sensitivity as gait monitoring insoles, where the stretchability of the material was not a primary concern. To verify its effectiveness, we designed a insole using the NCY‐TENG plain‐woven fabric. The design is illustrated in **Figure** **7**a,b, featuring an external circuit with a resistor for effective stress sensing. In constructing the insole, we carefully positioned stress sensing components in areas corresponding to key foot pressure points. For instance, we placed 15 stress sensing components, labeled S1 to S15, at specific locations such as the forefoot (S1), metatarsal (S2 to S7), arch (S8 to S11), and midfoot and heel regions (S12 to S15). Before finalizing the insole design, we examined the impact of external resistors on the output characteristics of the sensing component in the external circuit. The results indicated that the performance of the sensing fabric varied with the external resistor value, as shown in Figure S7 (Supporting Information). The highest output power (62.1 mW m^−2^) and a substantial output voltage (*V*_out_, 3.45 V) were achieved with an external resistance of 1 × 10^8^ Ω. Accordingly, this specific resistance was selected for the subsequent production of the gait monitoring insoles.

We further conducted experiments to evaluate the effectiveness of the gait monitoring insoles. In these experiments, a participant wore shoes fitted with these insoles while performing various standing and walking activities. The key objective was to understand how different gait conditions affect the pressure distribution on the foot sole, which then alters the *V*_out_ from each sensor. To accomplish this, we used the gait monitoring insoles to record *V*_out_ of each stress sensor under three distinct gait states: standard, out‐toeing, and in‐toeing gaits. This process involved mapping the stress values using the stress versus *V*_out_ characteristic curve (Figure S8, Supporting Information). Additionally, we used Ansys finite element analysis for simulations to visualize the pressure distribution on the sole, as shown in Figure 7c‐e. The simulations revealed key differences in pressure distribution among various gait types. For example, during a standard gait, the arch area experienced the least pressure, while the forefoot, metatarsal, and heel areas bore higher pressure. In contrast, the in‐toeing gait showed a higher‐pressure concentration on the outer side of the sole, whereas the out‐toeing gait had increased pressure on the inner side. In the context of gait analysis, it's important to note that the exact numerical measurements of force distribution across the foot sole's specific regions might not be crucial. The primary objective is to capture relative force distributions across different foot sole areas during activities such as walking or standing. This approach is adequate for identifying distinct gait patterns among individuals. Despite variations in speed and humidity within the footwear, these conditions uniformly affect all the fabric sensors embedded in the insole at any given moment, ensuring that the force‐output voltage relationship remains consistent across all sensors. This uniformity guarantees that, irrespective of changes in environmental factors, such as temperature, humidity, speed, or the patient's weight, the sensors accurately capture the foot sole's relative force distribution. This relative measurement approach provides reliable and sufficient data for analyzing gait patterns. These insights confirm the potential of gait monitoring insoles in providing accurate pressure mapping and identifying unique sole pressure characteristics associated with different gait states.

In addition to gait monitoring, NCY‐TENG plain‐woven fabrics offer several benefits for long‐term wear, particularly for individuals with conditions like diabetic foot. First, these fabrics exhibit outstanding breathability, a crucial comfort factor during extended wear. Breathability was assessed by measuring the gas flow rate through the fabrics under consistent size and pressure conditions (15 kPa), as depicted in Figure S9 (Supporting Information). NCY‐TENG fabrics have superior breathability compared to materials like electrospinning nanofiber membranes and coating films, and are slightly more breathable than cotton fabrics, s enhancing comfort, as shown in Figure 7f. Second, the antibacterial properties of these fabrics make them suitable for long‐term use, especially for diabetic foot patients who need a sanitized environment. This is achieved by incorporating Ag NWs in the adhesive layer of the fabrics, which have shown significant bactericidal characteristics. The antibacterial effectiveness was evidenced in colony counting experiments and OD value testing, as indicated in Figure 7g,h. These tests showed an 89% antibacterial rate against Gram‐negative bacteria and 84% against Gram‐positive bacteria. The effectiveness is mainly due to the Ag NWs in the adhesive layer. The Ag NWs integrated into the yarn are enveloped by a nanofiber layer that features a 3‐D, interconnected network structure. This configuration allows solvents, such as those from bacterial suspensions or sweat, to permeate the nanofiber layers and come into direct contact with the Ag NWs. In aquatic environments, especially those containing inorganic salts found in bacterial suspensions or sweat, the Ag NWs are capable of releasing silver ions. These ions are then able to migrate through the nanofiber layer, reach the yarn's surface, and exert their antibacterial effects by killing bacteria. Additionally, the hydrophobic nature of these fabrics imparts anti‐fouling properties, as shown in Figure S10 (Supporting Information). Furthermore, the NCY‐TENG plain‐woven fabrics exhibit remarkable durability, retaining ≈94% of their original output voltage even after 30 wash cycles. It should be noted that the PVA (model: 1795) used in the adhesive layer is of high molecular weight with a high alcoholysis degree (94%). They have extremely low solubility in water and can only dissolve in high temperatures, rendering it virtually insoluble in typical washing conditions. Coupled with their softness and light weight, this enhances the comfort for long‐term wear. Therefore, with attributes of breathability, antibacterial properties, hydrophobicity, durability, and comfort, NCY‐TENG plain‐woven fabrics are a highly promising material for gait monitoring, particularly for aiding diabetic foot patients.

## 3 Conclusion

In summary, this study presents the development of scalable, ultra‐sensitive, and stable core‐shell yarn‐based TENG textiles specifically designed for biomedical applications. By synergistically combining coaxial conjugated electrospinning with an on‐line conductive adhesive coating process, we fabricated NCYs by electrospinning PVDF and PA66 nanofibers onto stainless steel strands. These strands were pre‐coated with a PVA adhesive containing Ag NWs and liquid metals LM to enhance conductivity and functionality. Our NCYs exhibited exceptional qualities such as flexibility, mechanical strength, sewing capability, and braiding capacity, which make them suitable for various fabric designs. Moreover, employing flexible fabric structures like braiding, weaving, and knitting allowed the TENG textiles made from NCYs to demonstrate different levels of flexibility, stretchability, and sensing sensitivity. As a typical example, NCY‐TENG plain‐woven fabrics exhibit ultra‐high sensitivity (8.36 V kPa^−1^), a low limit of detection (0.01 kPa), excellent breathability, good anti‐bacterial, anti‐fouling, and washability. These were used for gait detection as demonstration. The developed NCYs and their derivative fabrics hold immense potential due to their scalable preparation, controllable structure, comfortable wearability, and functional expansibility. These textiles are not only suitable for real‐time monitoring of body motion signals such as gait and joint activity in diabetic foot patients, but also hold promising prospects in the broader field of wearable electronic devices.

## 4 Experimental Section

### Chemicals and Reagents

In this study, the PVDF used in our experiments refers to a copolymer consisting of vinylidene fluoride and trifluoroethylene. PVDF powder (PiezotechFC30) was obtained from Arkema S.A., France. PA66 particles (Zytel FG101L NC010, USA) were purchased from DuPont, USA. N, N dimethylformamide (DMF, ≥99.9%) and hexafluoroisopropanol (HFIP, 99.5%) were purchased from Aladdin Chemical Co., Ltd. Acetone was purchased from Sinopharm Chemical Reagent Co., Ltd., China. Stainless steel conductive yarns (316L) were obtained from Shengxin Special Rope Factory, China. The AgNWs dispersion (20 mg mL^−1^) was purchased from Nanjing XFNANO Materials Tech Co. Ltd., China. PVA (Mw = 89 000 to 98 000) was obtained from Sigma–Aldrich Chemical Co. Ltd. Carboxymethyl Cellulose (CMC, Mw = 250 000) was obtained from Aladdin Chemical Co., Ltd. Gallium (Mw = 69.72, 99.99%), Indium (Mw = 114.82, ≥99.9%), and Tin (Mw = 118.71, ≥99.9%) were purchased from Aladdin Chemical Co., Ltd. All materials were used as received without further purification.

### Preparation of Electrospinning Solutions

To prepare the electrospinning solutions of PVDF (10 wt.%), PVDF powder was dissolved in a mixture of DMF and acetone (mass ratio: 3:2) to a concentration of 10 wt.%. This mixture was stirred for 12 h at a temperature of 40 °C. Separately, the electrospinning solution of PA66 was prepared by dissolving PA66 particles in HFIP to a concentration of 10 wt.%. The mixture was stirred for 12 h at room temperature.

### Preparation of Conductive Adhesive

First, 0.02 g of CMC powder was dissolved in 8 mL of an AgNWs dispersion and stirred for 30 min at room temperature to form a dispersion of CMC/AgNWs. A mixture of 1 g of PVA powder and 12 mL of AgNWs dispersion was stirred for 1 h at 100 °C to form a dispersion of PVA/AgNWs. Gallium, indium, and tin particles were mixed in a mass ratio of 7:2:1, and stirred for 3 h at 300 °C to obtain liquid metal (LM). Subsequently, 1 g of LM was added to the CMC/AgNWs dispersion and then dispersed using an ultrasonic cell comminuter for 60 min, forming a LM/CMC/AgNWs suspension. Lastly, 6 mL of PVA/AgNWs dispersion was added to the above suspension and stirred for 15 min to obtain a uniformly mixed conductive adhesive suspension.

### Fabrication of PA66‐Based and PVDF‐Based NCYs

The continuous production of NCYs was achieved using a homemade coaxial conjugate electrospinning system (Figure 2a; Figure S1 and Movie S1, Supporting Information). The spinning system encompassed a coating container, two syringes equipped with propulsion pumps, two high‐voltage power supplies, a rotating metal funnel and a collection roller.

To electrospin the two types of NCYs, the following conditions were employed: First, an electrospinning solution was loaded into two separate 10 mL syringes equipped with 20 G metal nozzles. These syringes were positioned 6–10 cm away from the edge of the metal funnel. Second, the metal nozzles of the syringes were connected to positive and negative high‐voltage power supplies. During spinning, the solutions in the two syringes were attracted to each other due to the opposite high voltage, causing them to adhere to the rotating metal funnel and form a conical nanofiber web. Concurrently, a conductive stainless steel strand, acting as the core fiber, was coated with conductive adhesive and fed through the metal funnel. As the funnel rotated continuously, the core fibers were tightly enveloped by the nanofiber web, resulting in the formation of a yarn with a stable core‐shell structure. The NCYs were then collected continuously using a rotating roller.

The spinning parameters used were as follows: the electrospinning solution was fed at a rate of 2 mL h^−1^, while the metal funnel rotated at a speed of 300 rpm. The collection roller was set to rotate at a speed of 0–3 rpm. Additionally, the positive and negative voltages applied were ±7.5 kV for the PVDF electrospinning solution and ±5.2 kV for the PA66 electrospinning solution.

### Characterization and Measurement—Morphology Characterization

The surface and cross‐section morphology of the NCYs were characterized by the high‐resolution scanning electron microscopic (SEM, APREO S, thermo scientific, Netherlands).

### Characterization and Measurement—Adhesion Stability Test

The adhesion between the core fiber and the nanofiber shell layer of the yarn was evaluated using an electronic strength tester (YG004, Changzhou Xifang Instrument Co., Ltd., China). To conduct the test, the NCYs were initially cut into 10 mm segments. Subsequently, the shell layer of the yarn was secured by the upper fixture of the tester, while the lower fixture secured the core fiber. Two components of the yarn were then stripped at a 180° angle to determine the changes in adhesion between the two components during the stripping process.

### Characterization and Measurement—Output Performance of the Fabric

To quantitatively evaluate the electrical performance of yarns and fabrics, a custom‐built automatic circulation contact device with adjustable parameters (Figure 4a; Figures S3 and S4) was employed. The output voltage signals were measured using an oscilloscope (DSOX2014A, KEYSIGHT, USA) with an input impedance of 100 MΩ. Similarly, the output current signals and charge transfer were measured using an electrometer (6514, KEITHLEY, USA) with an ultra‐high load resistance of 200 TΩ.

### Characterization and Measurement—Characterization of Textile Properties

To evaluate the textile properties of the fabric, namely washability and breathability, specific procedures were followed. For assessing washability, a magnetic stirrer and a beaker containing deionized (DI) water were utilized to replicate a typical washing environment. The fabric underwent a 30‐min washing cycle, followed by drying at 60 °C for 1 h. The output voltage signals were then measured after complete drying.

In order to compare the breathability of the fabric with other triboelectric nanogenerators of traditional structure, a differential‐pressure method was employed. A breathability test device based on the differential‐pressure method was constructed for this purpose (Figure S7, Supporting Information). To facilitate a comparative analysis of breathability, a sandwich‐structured TENG was prepared by coating PVDF and PA66 films via electrostatic spinning. Additionally, the breathability of conventional cotton fabrics was also evaluated. The incoming airflow pressure was consistently maintained at 15 kPa throughout the testing process.

### Characterization and Measurement—Antibacterial Test

In this study, the antibacterial properties of various fabric samples were evaluated using absorbance value and spread plate methods against Gram‐negative E. coli and Gram‐positive S. aureus bacteria. All glassware, liquid medium, and experimental samples were sterilized in an autoclave at 0.1 MPa and 120 °C for 1 h prior to the experiment. The experimental samples consisted of i) NCY‐TENG plain‐woven fabric composed of PA66‐based and PVDF‐based NCYs with adhesive coating, ii) 12 cm core fiber with adhesive coating, and iii) control fabric composed of 6 cm PA66‐based and PVDF‐based NCYs without adhesive coating. For each sample, 1 mL of bacterial suspension (10^6^ CFU mL^−1^) was added to a separate well of a 24‐well cell culture plate, and the samples were completely immersed in the suspension. A blank group containing 1 mL of liquid medium without samples was used for reference. All samples were incubated in a constant temperature incubator at 37 °C for 16 h.

### Absorbance Value Method

After incubation, the bacterial suspensions from each sample were added to a 96‐well plate in 100 µL volumes. The absorbance values of each suspension were measured using an Elisa reader (SPARK, TECAN, Australia) at 600 nm. Three parallel samples were measured for each group, and the bacterial survival rate *R* was calculated using the following equation:

(2) $$R=\frac{OD_{S}-OD_{B}}{OD_{C}-OD_{B}}\times 100\%$$

where *OD*_S_ and *OD*_C_ were the absorbance values of the experimental and control groups, respectively, and *OD*_B_ was the absorbance value of the liquid medium without samples.

### Spread Plate Method

The bacterial suspension from each well was diluted 100 000 times, and 10 µL of the diluted suspension was inoculated onto the surface of a solid medium in a petri dish. These dishes were then incubated for 16 h at 37 °C. After incubation, individual bacteria grew and multiplied to form macroscopic colonies, which were counted to determine the number of bacteria in the original bacterial suspension. All procedures were performed under sterile conditions in a laminar flow hood.

## Conflict of Interest

The authors declare no conflict of interest.

## Supporting information

Supporting Information

Supplemental Movie 1

## Data Availability

The data that support the findings of this study are available from the corresponding author upon reasonable request.
