# Supplementary material for: Scalable and Ultra‐Sensitive Nanofibers Coaxial Yarn‐Woven Triboelectric Nanogenerator Textile Sensors for Real‐Time Gait Analysis
Source: Adv Sci (Weinh). 2024 May 15;11(28):2401436. doi: 10.1002/advs.202401436 (PMC11267306; doi:10.1002/advs.202401436)
Supplement: Supplementary file 1 — Supporting Information [file ADVS-11-2401436-s001.pdf]

ADVANCED SCIENCE
Open Access

Supporting Information

for *Adv. Sci.*, DOI 10.1002/advs.202401436

Scalable and Ultra-Sensitive Nanofibers Coaxial Yarn-Woven Triboelectric Nanogenerator Textile Sensors for Real-Time Gait Analysis

Yihan Wang, Lang Chu, Si Meng, Mingxuan Yang, Yidan Yu, Xiaokang Deng, Cheng Qi, Tiantian Kong* and Zhou Liu**

Supplementary Materials for

Scalable and ultra-sensitive nanofibers coaxial yarn-woven triboelectric nanogenerator textile sensors for real-time gait analysis

Yihan Wang et al.

*Corresponding author. E-mail: mengsi_simon@163.com; ttkong@szu.edu.cn; zhouliu@szu.edu.cn

This PDF file includes:

Figs. S1 to S10

Other Supplementary Materials for this manuscript include the following:

Movies S1

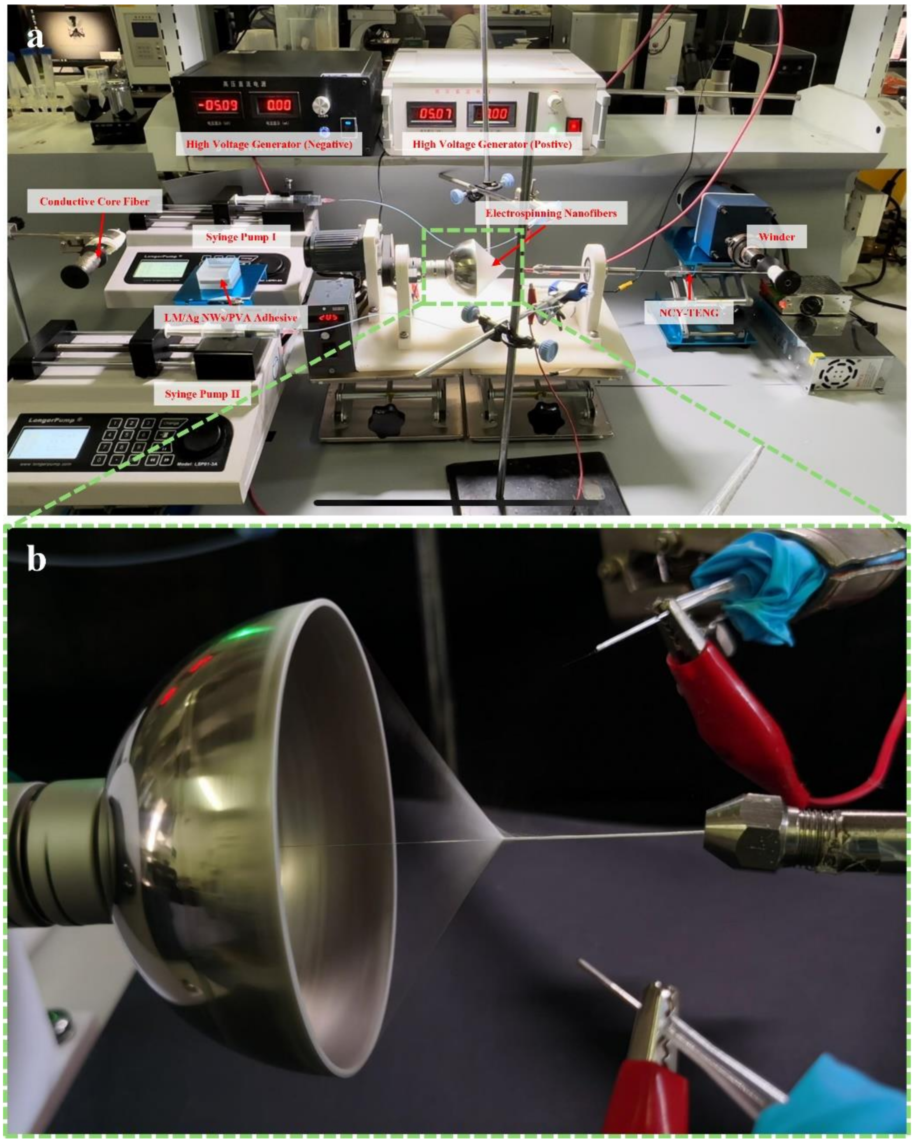

Figure. S1 Optical photograph of coaxial conjugate electrospinning device. (a) The overall perspective of coaxial conjugated electrospinning device; (b) A close-up of the component for wrapping nanofibers onto the core conductive fiber of NCY.

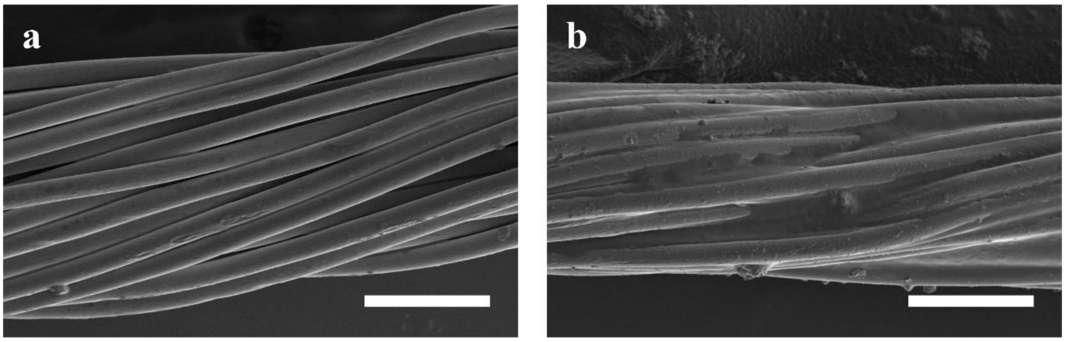

Figure. S2 SEM images of the core conductive fibers of NCY. (a) Core conductive fiber without adhesive; (b) Core conductive fiber with adhesive.

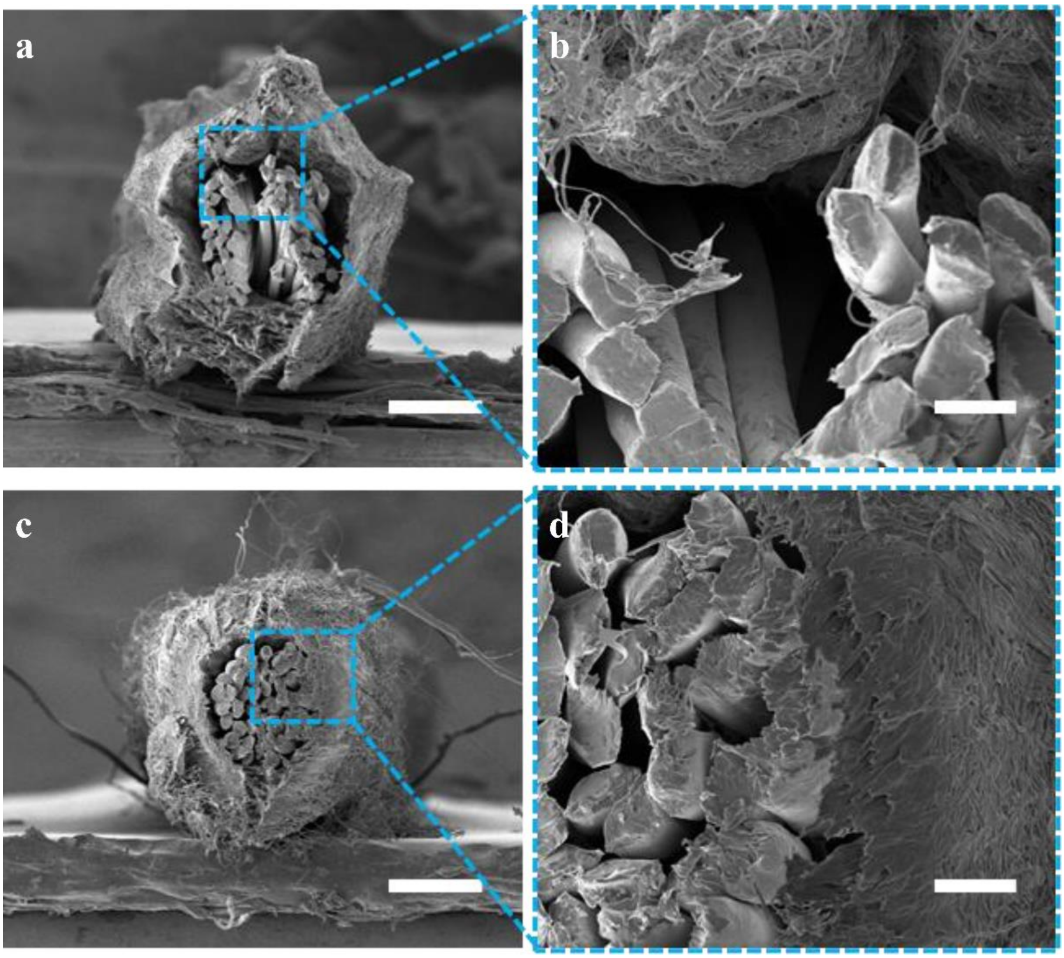

Figure. S3 SEM images of cross-section of PVDF-based NCYs with/without adhesive. (a) SEM images of cross-section of PVDF-based NCYs without adhesive, scale bar: 100 μm; (b) A partial magnification of cross-section of PVDF-based NCYs without adhesive, scale bar: 20 μm; (c) SEM images of cross-section of PVDF-based NCYs with adhesive, scale bar: 100 μm; (d) A partial magnification of cross-section of PVDF-based NCYs with adhesive, scale bar: 20 μm.

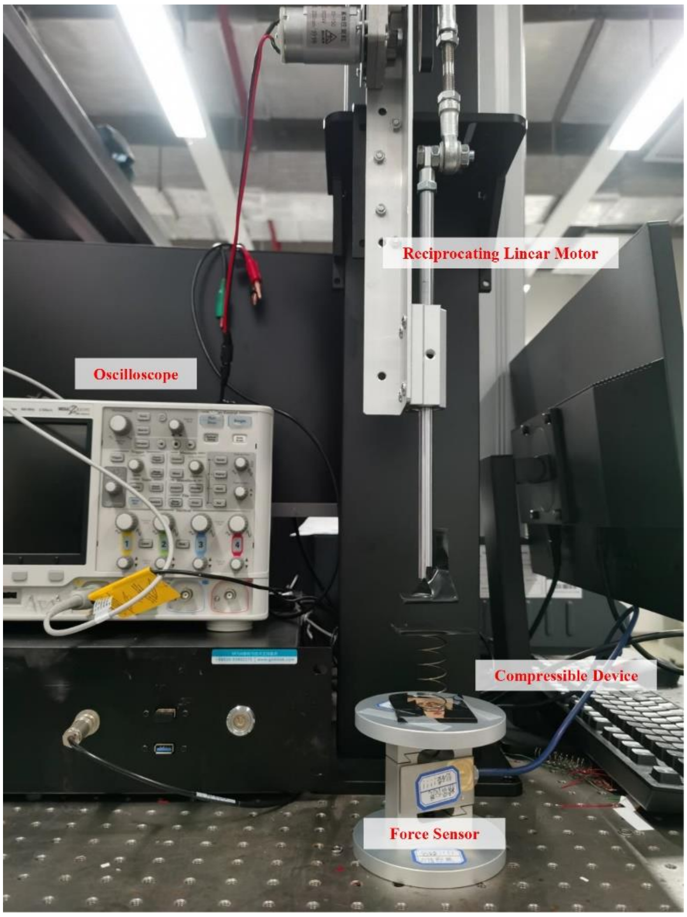

Figure. S4 Optical photograph of performance testing system for periodic stress sensing testing of PA66/PVDF-based NCY-TENG fabrics.

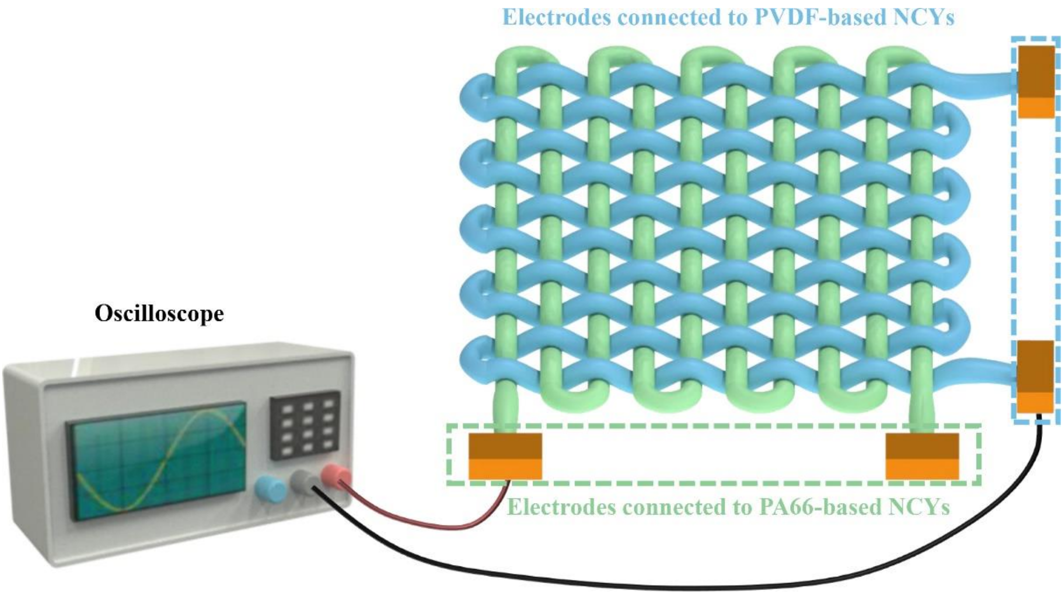

Figure. S5 Schematic diagram of component wiring for sensing performance testing of PA66/PVDF-based NCY-TENG fabrics.

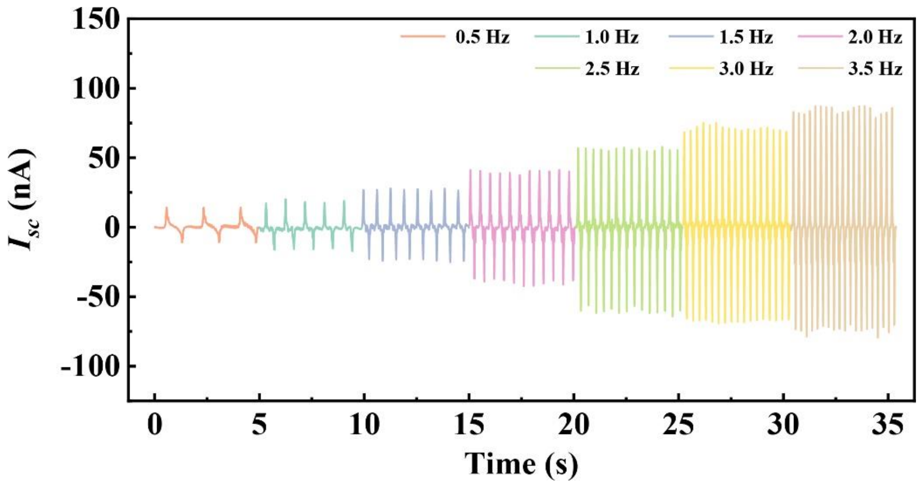

Figure. S6 Comparison of the short-circuit current generated by PA66/PVDF-based NCY-TENG plain-woven fabrics under 0.6 kPa pressure at different frequencies.

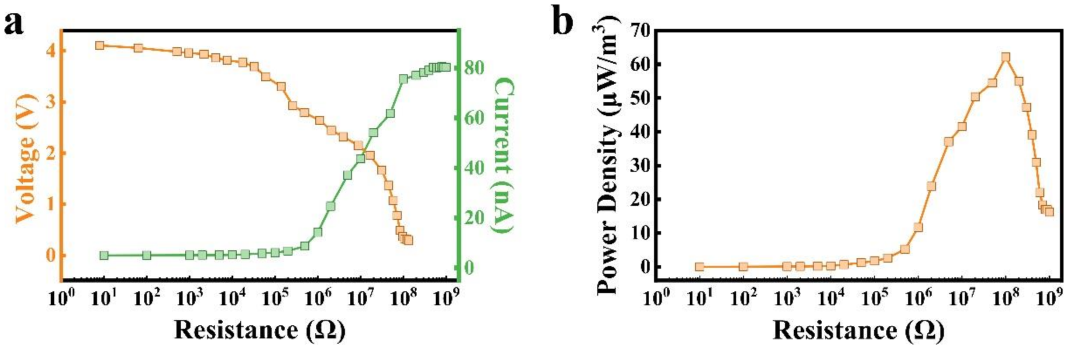

Figure. S7 The output voltage, output current, and output power density of the PA66/PVDF-based NCY-TENG plain-woven fabrics connected with resistors of different resistance values. (a) The output voltage and output current of the PA66/PVDF-based NCY-TENG plain-woven fabrics connected with resistors of different resistance values; (b) The output power density of the PA66/PVDF-based NCY-TENG plain-woven fabrics connected with resistors of different resistance values.

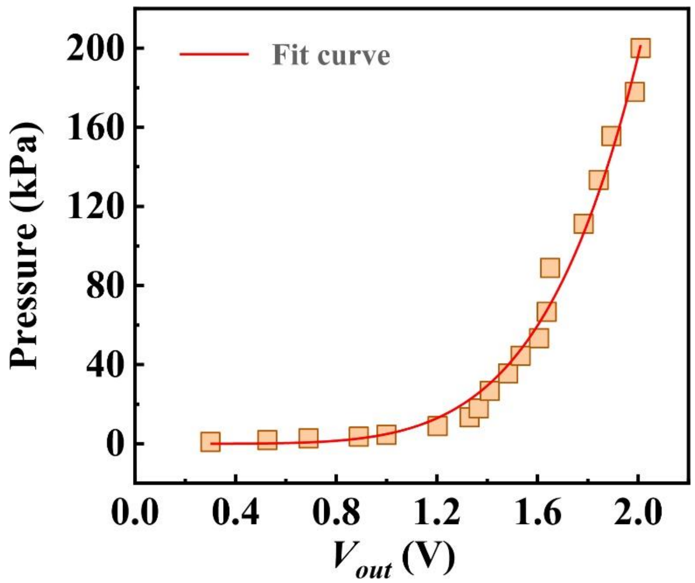

Figure. S8 Nonlinear fitting between the output voltage generated by the PA66/PVDF-based NCY-TENG plain-woven fabrics of the smart insole and the received pressure.

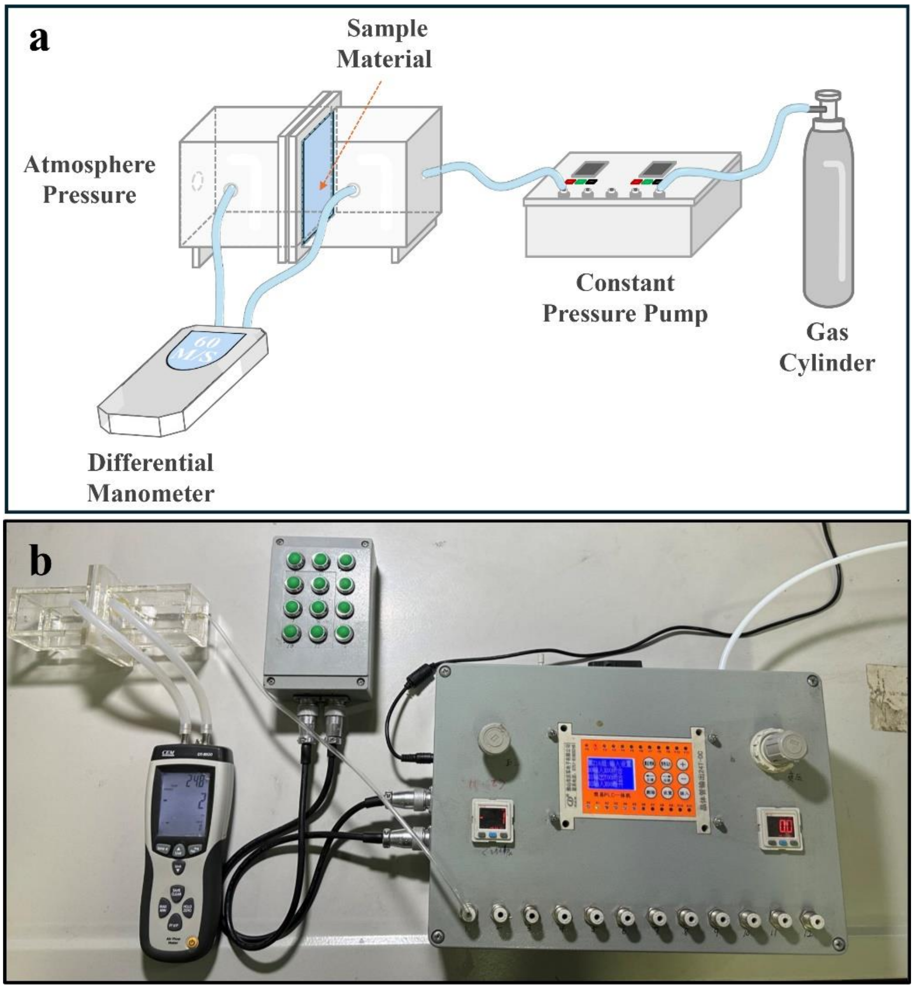

Figure. S9 Schematic diagram and optical photo of the fabric breathability testing system. (a) Schematic diagram of the fabric breathability testing system; (b) Optical photo of the fabric breathability testing system.

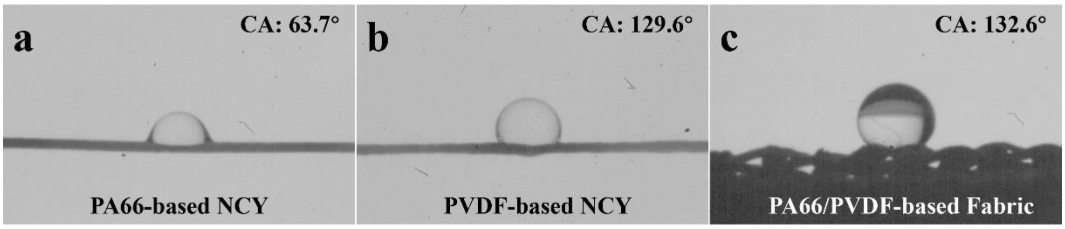

Figure. S10 Contact angle of water on PA66-based NCY, PAVDF-based NCY and PA66/PVDF-based NCY-TENG plain-woven fabric.

Movie S1.

This movie demonstrates the process of preparing nanofibers coaxial yarn through combining coaxial conjugated electrospinning and online conductive adhesive coating.
